# Nonporous Adaptive Crystals Based on Pillararene/Calixarene‐Inspired Novel Macrocyclic Arenes

**DOI:** 10.1002/EXP.20240286

**Published:** 2025-12-12

**Authors:** Susu Ren, Haitao Wang, Bowen Zha, Zhongping Li, Guan‐Yu Qiao, Jia‐Rui Wu

**Affiliations:** ^1^ Key Laboratory of Automobile Materials, MOE, School of Materials Science and Engineering Jilin University Changchun P. R. China; ^2^ Department of Chemical and Biomolecular Engineering Yonsei University Seodaemun‐gu Seoul Republic of Korea; ^3^ Department of Radiation Oncology China‐Japan Union Hospital of Jilin University Changchun P. R. China

**Keywords:** host–guest chemistry, molecular crystals, nonporous adaptive crystals, pillararene, supramolecular macrocycles

## Abstract

Nonporous adaptive crystals (NACs) represent a unique class of supramolecular macrocycle‐based crystalline organic materials that have garnered significant attention in supramolecular chemistry and beyond over the past decade. Unlike traditional porous materials, NACs are initially nonporous but can induce porosity through host–guest interactions in the solid‐state, enabling exceptional performance in hydrocarbon separation. This review surveys the development of NACs based on novel macrocyclic arenes inspired by pillararene and calixarene structures, encompassing biphen[n]arene, tiara[n]arene, leaning pillararene, hybrid[n]arene, leggero pillararene, geminiarene, bowtiearene, rhombicarene, and other derivatives. Emphasizing their preparation, structural characteristics, and mechanisms of adsorptive selectivity, this comprehensive overview highlights their contributions to advancing supramolecular chemistry, functional materials, and beyond. Finally, the remaining challenges and perspectives are outlined. It is anticipated that this review will serve as a timely and valuable reference for researchers interested in NACs and related materials, stimulating further impactful studies in related fields.

## Graphical abstract

Nonporous adaptive crystals (NACs), a novel class of macrocyclic receptor‐based adsorbent materials, have attracted considerable attention in supramolecular chemistry and beyond. This review surveys the development of NACs based on new macrocyclic arenes inspired by pillararene and calixarene structures, highlighting their preparation, structural characteristics, and adsorptive mechanisms from a crystal engineering viewpoint.

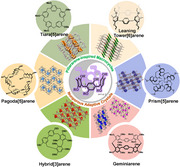

## 1 Introduction

Nonporous adaptive crystals (NACs), as a novel class of macrocyclic receptor‐based adsorbent materials, have garnered significant attention in the supramolecular community over the past decade [1, 2, 3, 4, 5, 6, 7, 8, 9, 10, 11, 12, 13, 14, 15, 16, 17, 18, 19, 20, 21, 22, 23, 24, 25, 26, 27, 28, 29, 30, 31, 32, 33, 34, 35, 36, 37, 38, 39, 40, 41, 42, 43, 44, 45, 46, 47, 48, 49, 50, 51, 52, 53, 54, 55, 56, 57, 58, 59, 60, 61, 62, 63, 64, 65, 66, 67, 68, 69, 70, 71, 72, 73, 74]. Unlike traditional porous materials such as zeolites [75, 76, 77], metal–organic frameworks (MOFs) [78, 79, 80, 81, 82, 83, 84, 85, 86, 87], and covalent organic frameworks (COFs) [88, 89, 90, 91, 92, 93, 94, 95, 96, 97], which possess rigid pore structures and high BET surface areas (typically exceeding 100 m^2^ g^−1^), NACs are nonporous in their initial state but exhibit structural adaptability. This unique feature allows for the induction of intrinsic or extrinsic porosity through weak intermolecular interactions with suitable guest molecules. As a result, NACs form host–guest assemblies in the solid state, accompanied by crystalline phase transformations [11, 31]. Remarkably, upon removal of complexed guest molecules through heating, NACs revert to their original nonporous forms, rendering them highly recyclable adsorbents. Moreover, NACs offer additional advantages such as ease of preparation, independence from crystallinity, and robust thermal and chemical resistance [36, 67].

The pursuit of macrocyclic arene‐based nonporous crystals can be traced back to the early 21st century, when Atwood and colleagues demonstrated that guest molecules could diffuse through the lattice of *p*‐tert‐butylcalix[4]arene crystals, despite the absence of molecular‐scale channels [98]. Subsequently, in 2015, Ogoshi et al. pioneered the study of pillararene crystals' adsorption properties towards various alkanes [33]. This milestone was followed by Huang and coworkers' conceptualization of NACs, ushering in a new era for the application of pillararene‐based materials in hydrocarbon separations of industrial relevance [31]. To date, pillararenes and their derivatives have proven effective in discriminating hydrocarbon mixtures that are challenging to separate via conventional distillation methods, including a wide range of compounds such as isooctane/*n*‐heptane [16], styrene/ethylbenzene [18], xylene isomers [27], and various positional and geometric isomers [9].

The pillararene family has consistently played a pivotal role in constructing supramolecular host‐based NACs since their inception [1, 2, 3, 4, 5, 6, 7, 8, 9, 10, 11, 12, 13, 14, 15, 16, 17, 18, 19, 20, 21, 22, 23, 24, 25, 26, 27, 28, 29, 30, 31, 32, 33]. The preparation methods, solid‐state host−guest chemistry, and diverse adsorptive separation applications of pillararene‐based NACs have been systematically summarized alongside their impressive research history [9, 31].

While traditional crystalline porous materials such as zeolites, MOFs, and COFs are known for their high surface areas and robust structures, they lack the inherent flexibility found in macrocyclic compounds like pillararenes and calixarenes. This flexibility, along with weak intermolecular interactions and excellent host–guest properties, enables NACs to adapt dynamically to guest molecules or external stimuli—an advantage that traditional porous materials cannot offer. Furthermore, the modular nature of these macrocycles allows for precise tuning of their properties, making them highly effective for selective adsorption and separation.

**SCHEME 1:**
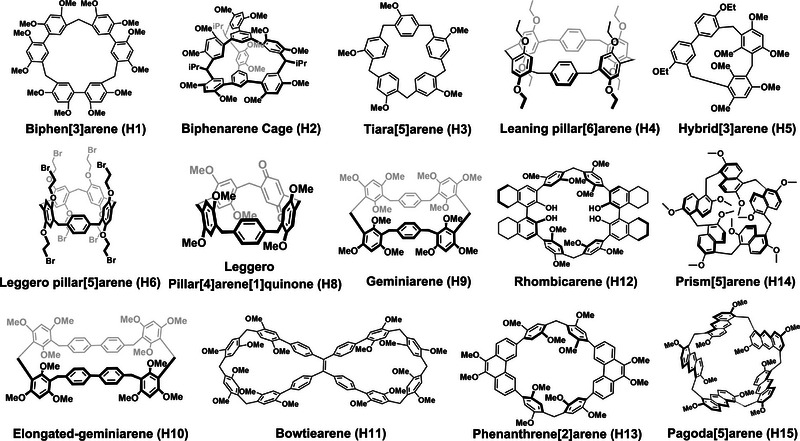
Chemical structures of the pillararene/calixarene‐inspired novel macrocyclic arenes discussed in this review.

To expand the toolkit of macrocycle‐based NACs and explore new frontiers in supramolecular chemistry, host–guest interactions, and functional materials, researchers have continuously unearthed and successfully introduced a series of new macrocycles inspired by pillararene and calixarene structures (Scheme 1). This review will provide an overview of research efforts in developing NACs based on these novel macrocyclic arenes, including biphenarene [56, 74], tiara[5]arene [60], hybrid[3]arene [70, 71, 72, 73], leaning tower[6]arene [58, 59], geminiarene [57], elongated‐geminiarene [61], bowtiearene [68], rhombicarene [62], leggero pillar[5]arene [67], phenanthrene[2]arene [63, 66], prism[5]arene [64] and pagoda[5]arene [69]. Emphasis will be placed on their structural characteristics, mechanisms of adsorptive selectivity, and the underlying principles of crystal engineering that govern their behavior. NACs constructed from small organic molecules and non‐pillararene/calixarene‐derived macrocycles and cages will not be covered here. Last, we outline future perspectives on the development of macrocyclic NACs, highlighting their potential contributions to advancing supramolecular chemistry and functional materials.

## 2 Biphen[n]Arene‐Based NACs

**FIGURE 1:**
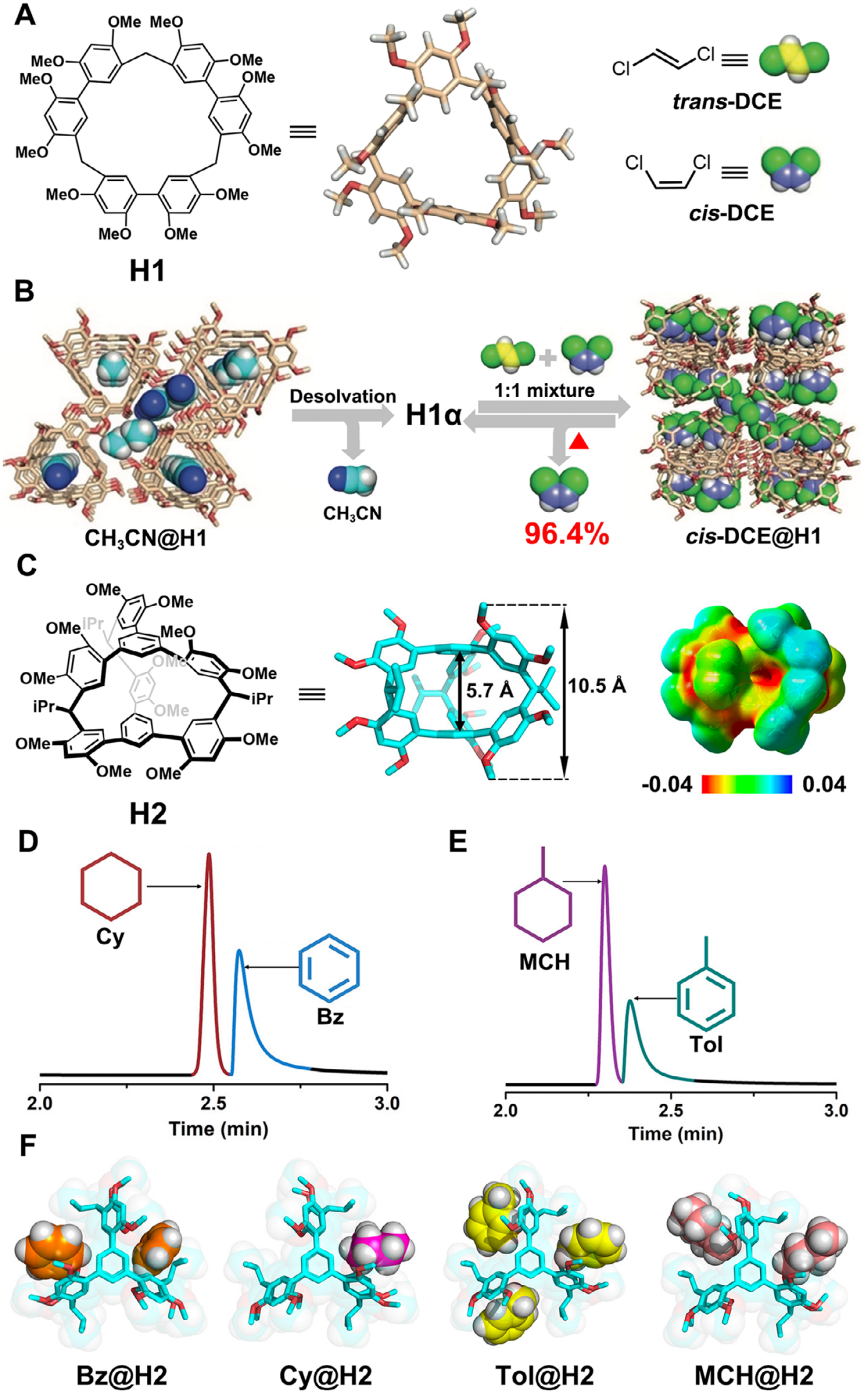
(A) Chemical structures of **H1**, ***trans*‐DCE**, and ***cis*‐DCE**. (B) Schematic representation of the selective separation of ***cis*‐DCE** from ***cis*‐DCE**/***trans*‐DCE** isomer mixture using **H1α**. Reproduced with permission [56]. Copyright 2019, John Wiley and Sons. (C) Chemical structures and electrostatic potential maps of biphenarene cage **H2**. **H2** coated column for GC separation of (D) **Cy**/**Bz** and (E) **Tol**/**MCH**. (F) Single‐crystal structures of **Bz**@**H2**, **Cy**@**H2**, **Tol**@**H2** and **MCH**@**H2**. Reproduced with permission [74]. Copyright 2021, John Wiley and Sons.

Biphen[n]arenes, pioneered by Li and coworkers in 2015, have gradually evolved into one of the largest families of macrocyclic arenes over the past decade [99, 100, 101, 102, 103, 104, 105, 106, 107]. They are distinguished by their modular synthetic strategy, customizable cavity sizes, diverse backbones, extensive binding capacity, and intriguing self‐assembly behavior. In 2019, they reported the first example of NACs based on biphen[3]arene, capable of separating industrially important *cis*‐/*trans*‐1,2‐dichloroethene (***cis*‐DCE** and ***trans*‐DCE**) isomers [56]. In this study, they first designed and synthesized 2,2′,4,4′‐tetramethoxylated biphen[3]arene (**H1**) with a triangular‐shaped topology in a near perfect yield of 99% (Figure 1A). Subsequently, NACs of **H1** (**H1α**) could be facilely prepared by desolvating the CH_3_CN‐loaded **H1** crystals (CH_3_CN**@H1)** under vacuum at 45°C. Single‐component solid‐vapor sorption experiments revealed that **H1α** could adsorb ***cis*‐DCE** vapor over time. As a contrast, the uptake of single‐component ***trans*‐DCE** vapor was negligible. When exposed to a 1:1 (v:v) mixture of ***cis*‐**/***trans*‐DCE** isomers vapor, **H1α** exclusively adsorbed ***cis*‐DCE** and underwent a phase transition to a ***cis*‐DCE**‐loaded form **(*cis*‐DCE@H1)** (Figure 1B). Upon heating, the complexed ***cis*‐DCE** molecules were released from ***cis*‐DCE@H1** with a purity of 96.4%, suggesting a good selectivity toward ***cis‐*DCE**. Furthermore, the removal of ***cis*‐DCE** restored the crystalline phase of ***cis*‐DCE@H1** to **H1α**, demonstrating the high recyclability of **H1**‐based NACs. This work represents a pioneering example of non‐pillararene‐based NACs for molecular separation.

Additionally, the scope of biphen[n]arenes‐based NACs was expanded from 2D macrocycles to 3D molecular cages by the same group in 2021 [74]. In this work, cage **H2** with a triangular prism‐shaped rigid structure and multiple π‐rich cavities was first designed and synthesized with a yield of 52% (Figure 1C). Then, NACs of **H2** (**H2α**) were prepared and demonstrated to serve as an effective gas chromatographic stationary phase for the separation of benzene (**Bz**)/ cyclohexane (**Cy**) and toluene (**Tol**)/ methylcyclohexane (**MCH**) (Figure 1D,E). Single crystal structure analysis revealed that all guest molecules were complexed in the external cavities of **H2** and/or channels between adjacent **H2** molecules (Figure 1F), and the effective separation could be ascribed to the different host–guest binding modes and stabilities among the **H2** crystals loaded with the four different guests.

## 3 Tiara[5]Arene‐Based NACs

**FIGURE 2:**
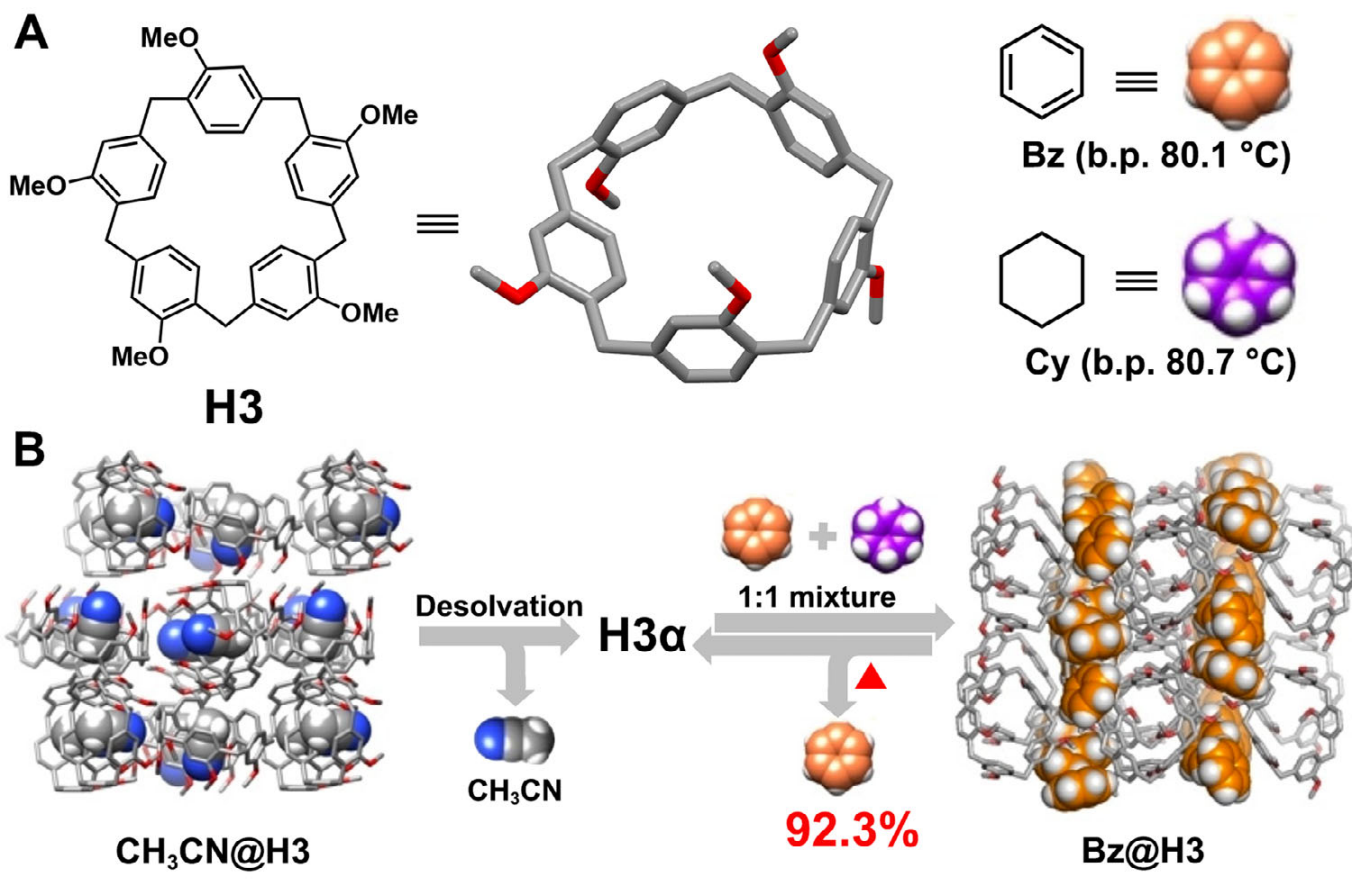
(A) Chemical structures of **H3**, **Bz**, and **Cy**. (B) Schematic illustration of the separation of **Bz** and **Cy** using **H3α**. Reproduced with permission [60]. Copyright 2020, John Wiley and Sons.

Tiara[n]arenes, first introduced by Sue and coworkers in 2019, are a class of new macrocyclic arenes derived from pillar[n]arenes [60]. Unlike traditional synthetic routes involving phenol‐formaldehyde condensation, tiara[n]arenes are composed of five or six phenolic units *para*‐bridged by methylenes, and are obtained through the selective hydrodeoxygenation of pillar[n]arene derivatives with rim‐differentiated features [108, 109]. Sue et al. demonstrated the highly selective separation of benzene (**Bz**) from cyclohexane (**Cy**) using NACs based on permethylated tiara[5]arene (**H3**) (Figure 2A). Upon exposure to a 1:1 (v:v) mixture of **Bz**/**Cy** vapor, adaptive **H3** crystals (**H3α**) exclusively adsorbed **Bz**, leading to a structural transformation into a **Bz**‐loaded form (**Bz@H3**) (Figure 2B). Single crystal structure analysis revealed that **Bz** molecules were stabilized in the channels between stacks of **H3** via C‐H···O and C‐H···π interactions. Gas chromatography (GC) confirmed that **Bz** purity reached up to 92.3% upon release from **Bz@H3**, indicating the high selectivity of **H3α** towards **Bz**. Furthermore, the removal of **Bz** restored the phase of **Bz@H3** back to **H3α**, enabling multiple cycles of reuse without performance degradation.

## 4 Leaning Pillar[6]Arene‐Based NACs

In 2018, Wu and Yang designed and synthesized a novel macrocyclic host, named leaning pillar[6]arene, which was designated according to its inclined molecular conformation in the solid state compared to traditional pillar[6]arene [110]. Leaning pillar[6]arene not only represents a new class of macrocyclic receptor derived from phenol‐formaldehyde synthesis chemistry but also serves as a “defunctionalized” version of pillar[6]arene, where functional groups on one pair of opposite benzene rings of the traditional pillar[6]arene backbone have been selectively replaced with hydrogen atoms.

**FIGURE 3:**
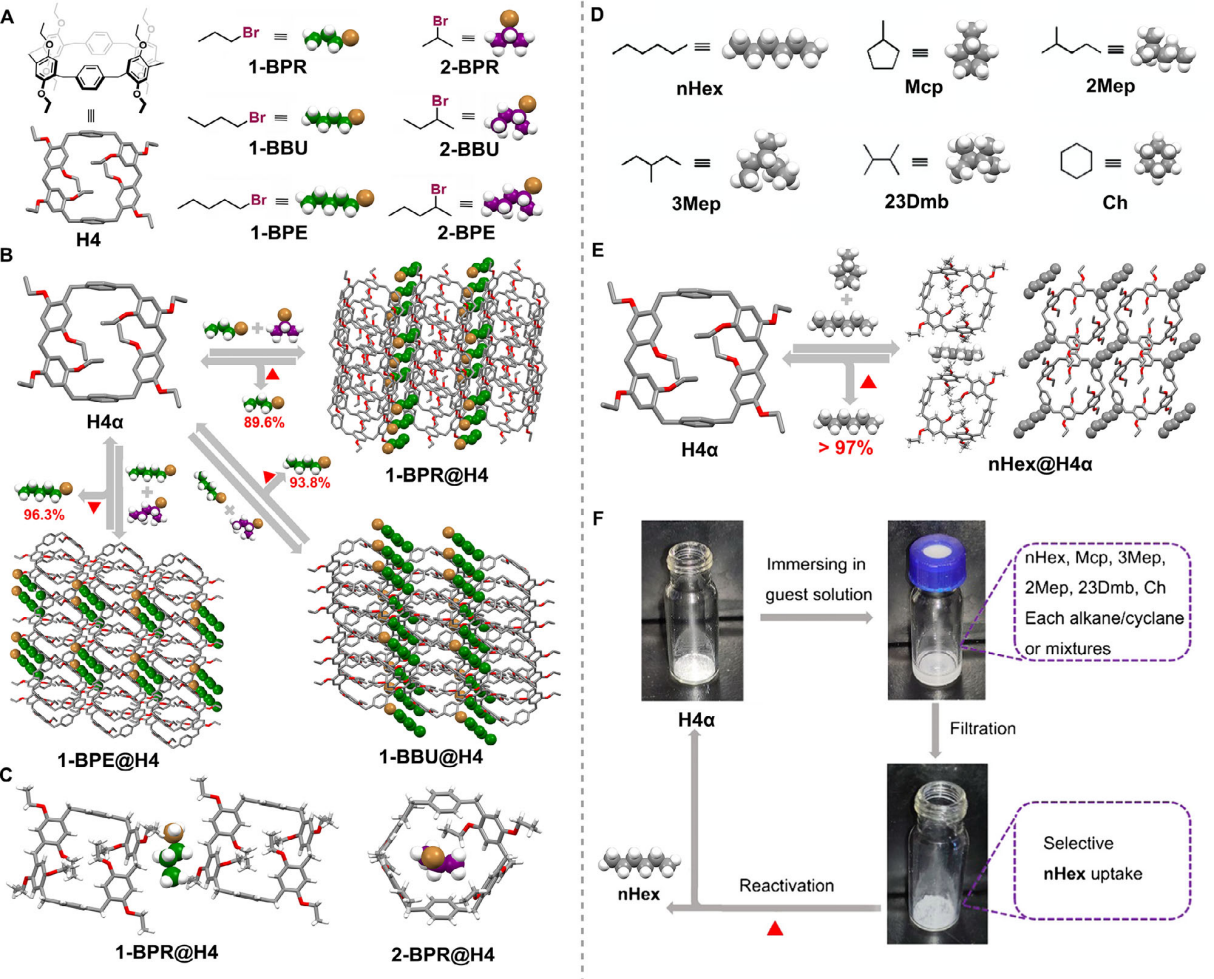
(A) Chemical structures of the macrocyclic host **H4** and **1‐BPR**, **2‐BPR**, **1‐BBU**, **2‐BBU**, **1‐BPE** and **2‐BPE**. Note that **2‐BBU** and **2‐BPE** are chiral, and a racemic mixture (R/S) was used in this study. (B) Schematic representation of the selective separation of 1‐bromoalkanes from 1‐/2‐bromoalkane isomers using **H4α**. (C) Single‐crystal structures of **1‐BPR@H4** and **2‐BPR@H4**. Reproduced with permission [58]. Copyright 2020, John Wiley and Sons. (D) Chemical structures of selected C6 alkanes/cycloalkanes. (E) Schematic illustration of the separation of **nHex** and **Mcp** using **H4α**. (F) Schematic illustration of the solid–liquid uptake method for **nHex** purification. Reproduced with permission [59]. Copyright 2021, Chinese Chemical Society.

By virtue of the superior structural flexibility and cavity adaptability of leaning pillar[6]arene, they successfully constructed NACs based on perethylated leaning pillar[6]arene (**H4**) in 2020 and applied it for the selective adsorption separation of 1‐/2‐bromoalkanes isomers (Figure 3A) [58]. In this work, NACs of **H4** (**H4α**) selectively adsorbed 1‐bromoalkanes over its 2‐ positional isomers upon exposure to 1:1 (v:v) mixtures of 1‐bromopropane (**1‐BPR**)/ 2‐bromopropane (**2‐BPR**), 1‐bromobutane (**1‐BBU**)/ 2‐bromobutane (**2‐BBU**) and 1‐bromopentane (**1‐BPE**)/ 2‐bromopentane (**2‐BPE**), respectively, accompanied by the phase transitions to the 1‐bromoalkanes‐loaded forms (**1‐BPR@H4**, **1‐BBU@H4**, and **1‐BPE@H4**) (Figure 3B). Upon heating, 1‐bromoalkane molecules could be released from **1‐BPR@H4**, **1‐BBU@H4**, and **1‐BPE@H4** with purities of 89.6%, 93.8%, and 96.3%, respectively, indicating remarkable selectivity for 1‐bromoalkanes. Single crystal structure combined with powder X‐ray diffraction (PXRD) analyses revealed that this selectivity stemmed from the different host–guest interactions and thermal stabilities between the **H4** crystals loaded with 1‐bromoalkanes and 2‐bromoalkanes. Specifically, the 1‐bromoalkane molecules in **1‐BPR@H4**, **1‐BBU@H4**, and **1‐BPE@H4** were located in the external pore space between adjacent **H4** molecules, contrasting with the inclusion complexes formed by the 2‐bromoalkane molecules and **H4** (typified by **2‐BPR@H4**) (Figure 3C). Moreover, removing the complexed 1‐bromoalkanes transformed the crystalline phases of 1‐bromoalkane‐loaded crystals back to **H4α**, allowing for multiple reuse cycles without loss of performance.

Based on the above work, we further investigated the separation of n‐hexane (**nHex**) and methylcyclopentane (**Mcp**) using **H4α** (Figure 3D) [59]. **H4α** effectively adsorbed **nHex** from a 1:1 (v:v) mixture of **nHex**/**Mcp** with a purity exceeding 97%, employing lossless solid‐vapor adsorption and high‐speed solid–liquid adsorption methods (Figure 3E). Similar to the structures loaded with 1‐bromoalkanes, the complexed **nHex** molecules were positioned in the extrinsic pores formed by the adjacent **H4** molecules through stabilization via C─H···π interactions. Importantly, **H4α** maintained its selectivity for **nHex** even when exposed to mixtures containing other C6 alkanes/cycloalkanes, including 2‐methylpentane, 3‐methylpentane, 2,3‐dimethylbutane, and cyclohexane (Figure 3D,F), underscoring the practical utility of **H4α** for **nHex** purification.

## 5 Hybrid[3]Arene‐Based NACs

Hybrid[n]arenes, are a class of new macrocyclic arenes derived from pillar[n]arenes and/or calix[n]arenes, combining two or more distinct aromatic units within a single molecule [111, 112, 113, 114, 115, 116]. These supramolecular hosts are characterized by their “hybrid” nature, which provides them with versatility in design and potential for tunable properties.

**FIGURE 4:**
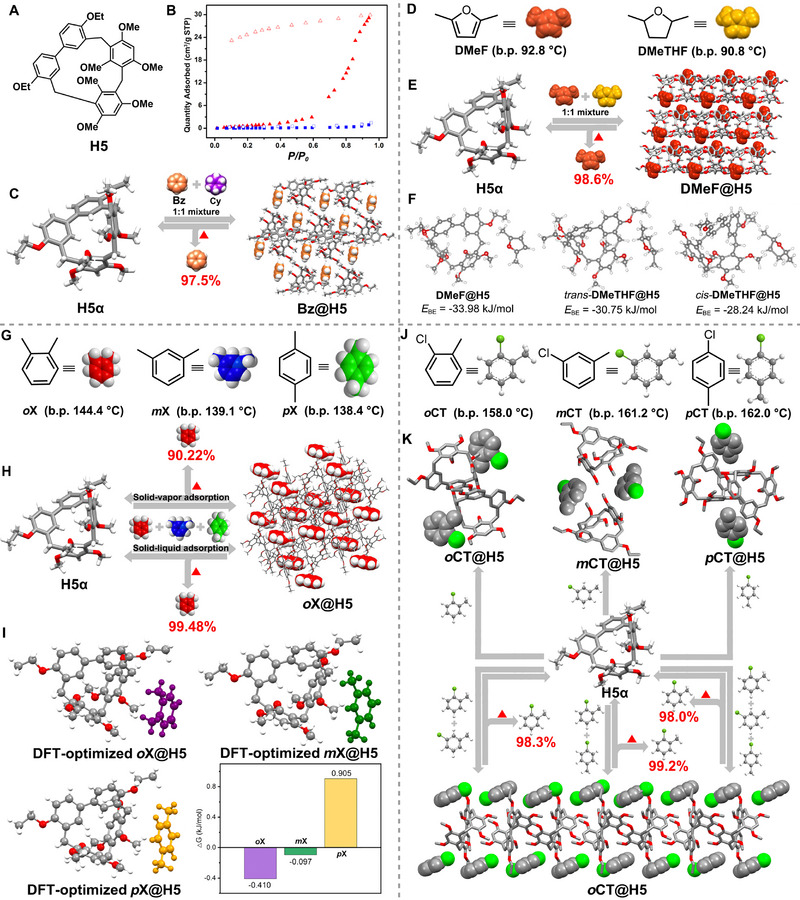
(A) Chemical structure of **H5**. (B) Vapor sorption isotherms of **H5α** toward **Bz** (red triangles) and **Cy** (blue squares). (C) Schematic illustration of the separation of **Bz** and **Cy** using **H5α**. Reproduced with permission [70]. Copyright 2020, American Chemical Society. (D) Chemical structures and cartoon representations of **DMeF** and **DMeTHF**. Note that the stereochemistry of **DMeTHF** is ambiguous, and a mixture of cis and trans isomers was used in this study. (E) Schematic illustration of the separation of **DMeF** and **DMeTHF** using **H5α**. (F) Calculated binding energies (*E*_BE_) between **H5** and **DMeF**, *trans*‐**DMeTHF**, and *cis*‐**DMeTHF**. Reproduced with permission [71]. Copyright 2023, Royal Society of Chemistry. (G) Chemical structures and cartoon representations of ***o*X**, ***m*X** and ***p*X**. (H) Schematic illustration of the selective separation of xylene isomers using **H5α**. (I) The DFT‐optimized structures and calculated binding energies for ***o*X@H5**, ***m*X@H5**, and ***p*X@H5**. Reproduced with permission [72]. Copyright 2024, Royal Society of Chemistry. (J) Chemical structures and cartoon representations of ***o*CT**, ***m*CT**, and ***p*CT**. (K) Schematic illustration of the selective separation of monochlorotoluene isomers using **H5α**. Reproduced with permission [73]. Copyright 2024, American Chemical Society.

In 2016, Yu and coworkers reported the synthesis of the first type of hybrid[3]arene (**H5**) comprising one 4,4′‐biphenol diethyl ether unit and two 1,3,5‐trimethoxybenzene units (Figure 4A) [115]. Later, in 2019, they introduced the first example of **H5**‐based NACs (**H5α**) capable of separating benzene (**Bz**) and cyclohexane (**Cy**) [70]. Vapor sorption isotherms of **H5α** toward single‐component **Bz** and **Cy** demonstrated its ability to adsorb **Bz** above a critical vapor pressure (*P*/*P*_0_ = 0.6), showing gate‐opening behavior (Figure 4B). In contrast, minimal **Cy** adsorption occurred even at a higher pressure (*P*/*P*_0_ = 1.0). Upon exposure to a 1:1 (v:v) **Bz** and **Cy** mixture, **H5α** selectively adsorbed **Bz**, transforming into a **Bz**‐loaded form (**Bz@H5**) (Figure 4C). Similar to the previously discussed **H4** host, the adsorbed **Bz** molecules were positioned in the self‐assembled extrinsic pores and sandwiched between two adjacent **H5** hosts through stable C─H···π and C─H···O interactions. The selectivity of **H5α** was attributed to forming a highly stable crystalline phase upon preferential **Bz** uptake. Upon heating, **Bz** molecules could be released from **Bz@H5** with a purity of 97.5%, and the original **H5α** could be recovered and reused many times without degradation.

In 2023, Wu and coworkers investigated the separation of 2,5‐dimethylfuran (**DMeF**) and 2,5‐dimethyltetrahydrofuran (**DMeTHF**) using **H5α** NACs (Figure 4D) [71]. Solid–vapor adsorption experiments confirmed that **H5α** could separate **DMeF** from a 1:1 (v:v) **DMeF**/**DMeTHF** mixture with 98.6% purity, accompanied by the phase transition from **H5α** to a **DMeF**‐loaded form (**DMeF@H5**) (Figure 4E). Single crystal structure analysis combined with theoretical calculations indicated this selectivity stemmed from forming a highly stable crystal structure upon capturing preferred guest **DMeF** (Figure 4F).

Shortly after, Zhou and coworkers demonstrated the efficacy of **H5α** in selectively separating xylene isomers (Figure 4G) [72]. Specifically, **H5α** effectively separated *ortho*‐xylene (***o*X**) from a 1:1:1 (v:v) mixture of ***o*X**/meta‐xylene (***m*X**)/para‐xylene (***p*X**) using both solid–vapor and solid–liquid phase adsorption approaches, achieving purities of 90.22% and 99.48%, respectively (Figure 4H). The selectivity of **H5α** for ***o*X** was attributed to forming a stable ***o*X**‐loaded crystal structure (***o*X@H5**) upon capturing the preferred guest, facilitated by specific C─H···π and C─H···O interactions between **H5α** and ***o*X**. Moreover, theoretical calculations showed that the adsorption energy of **H5** toward ***o*X** was the smallest among the xylene isomers, further confirming the preferred adsorption of **H5α** for ***o*X** (Figure 4I).

Almost simultaneously, Zhou and coworkers also investigated the selective separation of monochlorotoluene isomers using **H5α** NACs (Figure 4J) [73]. **H5α** effectively adsorbed single‐component *ortho*‐chlorotoluene (***o*CT**), *meta*‐chlorotoluene (***m*CT**), and *para*‐chlorotoluene (***p*CT**) vapors, resulting in phase transitions to ***o*CT**, ***m*CT**, and ***p*CT**‐loaded forms (***o*CT@H5**, ***m*CT@H5**, and ***p*CT@H5**), respectively (Figure 4K). Notably, **H5α** exhibited strong selectivity for ***o*CT** over other monochlorotoluene isomers, achieving purities of 98.3%, 99.2%, and 98.0% from equimolar mixtures of ***o*CT**/***m*CT**, ***o*CT**/***p*CT**, and ***o*CT**/***m*CT**/***p*CT**, respectively. Similarly, this selectivity was attributed to differing binding modes and stabilities among the ***o*CT@H5α**, ***m*CT@H5α**, and ***m*CT@H5α** crystals. Above all, NACs of **H5** exhibit significant promise for practical applications in the adsorptive separation of petrochemical feedstocks.

## 6 Leggero Pillar[5]Arene‐Based NACs

**FIGURE 5:**
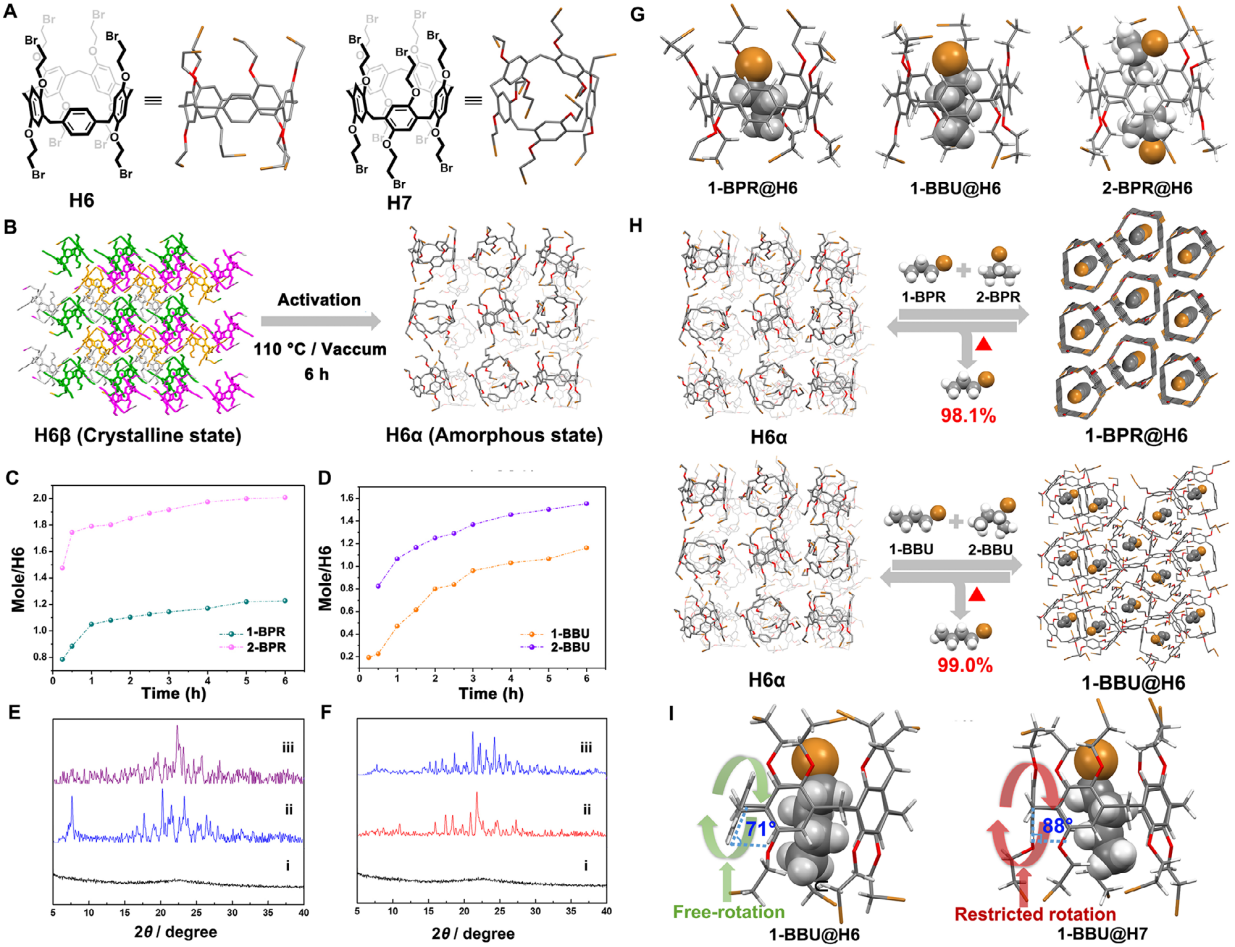
(A) Chemical structures of **H6** and **H7**. (B) Schematic representation of the phase transformation from crystalline **H6β** to amorphous **H6α** by heating in vacuum. Time‐dependent **H6α** solid‐vapor adsorption plots for single‐component (C) **1‐BPR** and **2‐BPR** vapors and (D) **1‐BBU** and **2‐BBU** vapors. (E) PXRD patterns of **H6**: (i) **H6α**, after adsorption of (ii) **2‐BPR** vapor and (iii) **1‐BPR** vapor, suggesting the formation of two new guest‐loaded **H6** structures **2‐BPR@H6** and **1‐BPR@H6**. (F) PXRD patterns of **H6**: (i) **H6α**, after adsorption of (ii) **2‐BBU** vapor and (iii) **1‐BBU** vapor, suggesting the formation of two new guest‐loaded **H6** structures **2‐BBU@H6** and **1‐BBU@H6**. (G) Single crystal structures of **1‐BPR@H6**, **1‐BBU@H6**, and **2‐BPR@H6**. (H) Schematic representation of the selective separation of 1‐bromoalkanes from 1‐/2‐bromoalkane isomers using **H6α**. (I) Comparison of the crystal structures between **1‐BBU@H6** and **1‐BBU@H7**. Reproduced with permission [67]. Copyright 2022, AAAS.

Leggero pillar[n]arenes are a class of novel macrocyclic arenes derived from pillar[n]arenes, first introduced by Wu and Yang in 2021 [117]. They are characterized by a structural feature that includes one unsubstituted phenylene unit, distinguishing them from traditional pillar[n]arenes. Later in 2022, they reported the first example of NACs based on leggero pillar[5]arene capable of separating 1‐/2‐bromoalkane isomers [67]. In this study, perbromoethylated leggero pillar[5]arene (**H6**), bearing eight bromoethyl moieties, was designed and synthesized (Figure 5A). Activated adsorptive separation materials (**H6α**) were obtained by heating the guest‐free and thermodynamically favored **H6** crystals (**H6β**) under vacuum at 110°C (Figure 5B). Interestingly, PXRD revealed that **H6α** exhibited an amorphous phase, indicating the collapse of the well‐ordered molecular arrangement of **H6β** upon heating. Single‐component solid–vapor sorption experiments confirmed that **H6α** could adsorb both 1‐bromoalkanes (**1‐BPR** and **1‐BBU**) and 2‐bromoalkanes (**2‐BPR** and **2‐BBU**) (Figure 5C,D), leading to structural transformations from amorphous **H6α** to four different crystalline host–guest assemblies referred to as **1‐BPR@H6**, **1‐BBU@H5**, **2‐BPR@H6**, and **2‐BBU@H5**, respectively (Figure 5E,F). Different from the exo‐wall binding observed in leaning pillar[6]arene (**H4**) loaded with 1‐bromoalkanes, **H6** binds both 1‐/2‐bromoalkanes isomers through in‐cavity host–guest interactions. In the structures of **1‐BPR@H6** and **1‐BBU@H5**, one 1‐bromoalkane molecule is stably encapsulated in the **H6** cavity via multiple C─H···π, C─H···O, and C─H···Br interactions. By contrast, **2‐BPR** molecules in **2‐BPR@H6** are irregularly located at the cavity rims of **H6**, displaying a structural mismatch with the linear cavity due to its branched structure (Figure 5G). As anticipated, **H6α** selectively adsorbed **1‐BPR** and **1‐BBU** when exposed to the mixtures (v:v = 1:1) of **1‐BPR/2‐BPR** and **1‐BBU/2‐BBU**, resulting in guest‐induced amorphous‐to‐crystalline phase transformations from **H6α** to **1‐BPR@H6** and **1‐BBU@H5**, respectively (Figure 5H). GC analysis confirmed purities of 98.1% and 99.0% for adsorbed **1‐BPR** and **1‐BBU** in **H6**, highlighting the remarkable selectivity of **H6** for 1‐bromoalkane isomers. Controlled experiments demonstrated that **H6** exhibited better adsorptive selectivity toward 1‐/2‐bromoalkane mixtures compared to its counterpart macrocyclic arene, perbromoethylated pillar[5]arene (**H7**), derived from traditional pillararenes (Figure 5I). Single‐crystal structure analysis combined with theoretical calculations demonstrated that this improved sorting ability could be attributed to the superior cavity adaptability and structural flexibility of **H6**, facilitated by the free‐rotation/unsubstituted phenylene subunit on its backbone.

**FIGURE 6:**
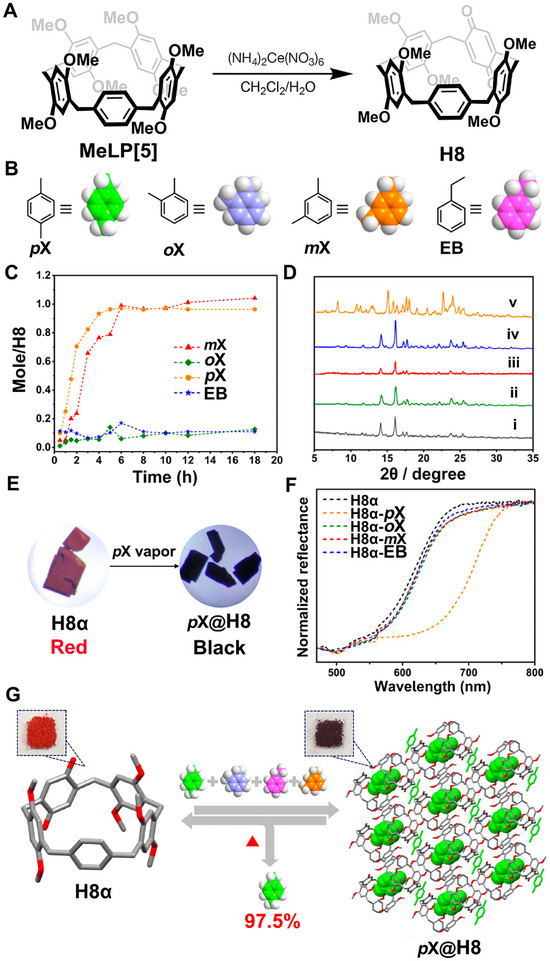
(A) Synthetic route to **H8**. (B) Chemical structures and cartoon representations of C8 alkylaromatics, that is ***o*X**, ***m*X**, ***p*X**, and **EB**. (C) Time‐dependent **H8α** solid–vapor adsorption plots for single‐component ***o*X**, ***m*X**, ***p*X**, and **EB** vapors. (D) PXRD patterns of **H8**: (i) **H8α**; after adsorption of (ii) ***o*X**, (iii) ***m*X**, (iv) **EB**, and (v) ***p*X**. (E) Photographs of **H8α** before and after adsorption of ***p*X**. (F) Diffuse reflectance spectra of **H8α** after exposure to ***o*X**, ***m*X**, ***p*X**, and **EB** vapors, respectively. (G) Schematic representation of the transformation from **H8α** to ***p*X@H8** along with the color change from red to brown upon uptake of the ***o*X**/***m*X/*p*X**/**EB** mixture vapor. Reproduced with permission [118]. Copyright 2024, American Chemical Society.

Recently, Yang and coworkers reported another example of NACs based on leggero pillar[5]arene capable of separating *p*X from C8 alkylaromatics with vapochromic behaviors (Figure 6A,B) [118]. In this study, a new derivative of Leggero pillar[5]arene containing one benzoquinone unit, leggero pillar[4]arene[1]quinone (**H8**), was synthesized by partial oxidation of permethylated leggero pillar[5]arene (**MeLP[5]**) using ammonium cerium nitrate (Figure 6A). Subsequently, NACs of **H8** (**H8α**) with red color were prepared using a simple recrystallization and desolvation approach. Single‐component solid–vapor sorption experiments revealed that both ***p*X** and ***m*X** could be absorbed by **H8α** (Figure 6C), but only ***p*X** could induce a guest‐loaded crystal‐to‐crystal transformation from **H8α** to a ***p*X**‐loaded form (***p*X@H8**) (Figure 6D), accompanied by a color change from red to black (Figures 6E,F). In contrast, the uptake of single‐component ***o*X** and ethylbenzene (**EB**) vapor was negligible. When exposed to an equal volume mixture of C8 aromatics, **H8α** selectively adsorbed ***p*X** over others with a purity exceeding 97% along with a distinct color change, enabling simultaneous separation and detection of ***p*X** (Figure 6G). Single‐crystal structure analysis combined with PXRD confirmed that the selectivity was derived from the formation of a highly stable crystal structure upon capturing the preferred guest ***p*X**, and the color change stemmed from the distinct charge transfer interactions between adjacent **H8** hosts in **H8α** and ***p*X@H8**. Moreover, the removal of ***p*X** restored the crystalline phase and the red color of **H8α**, making these crystals highly recyclable without loss of performance.

## 7 Geminiarene‐Based NACs

**FIGURE 7:**
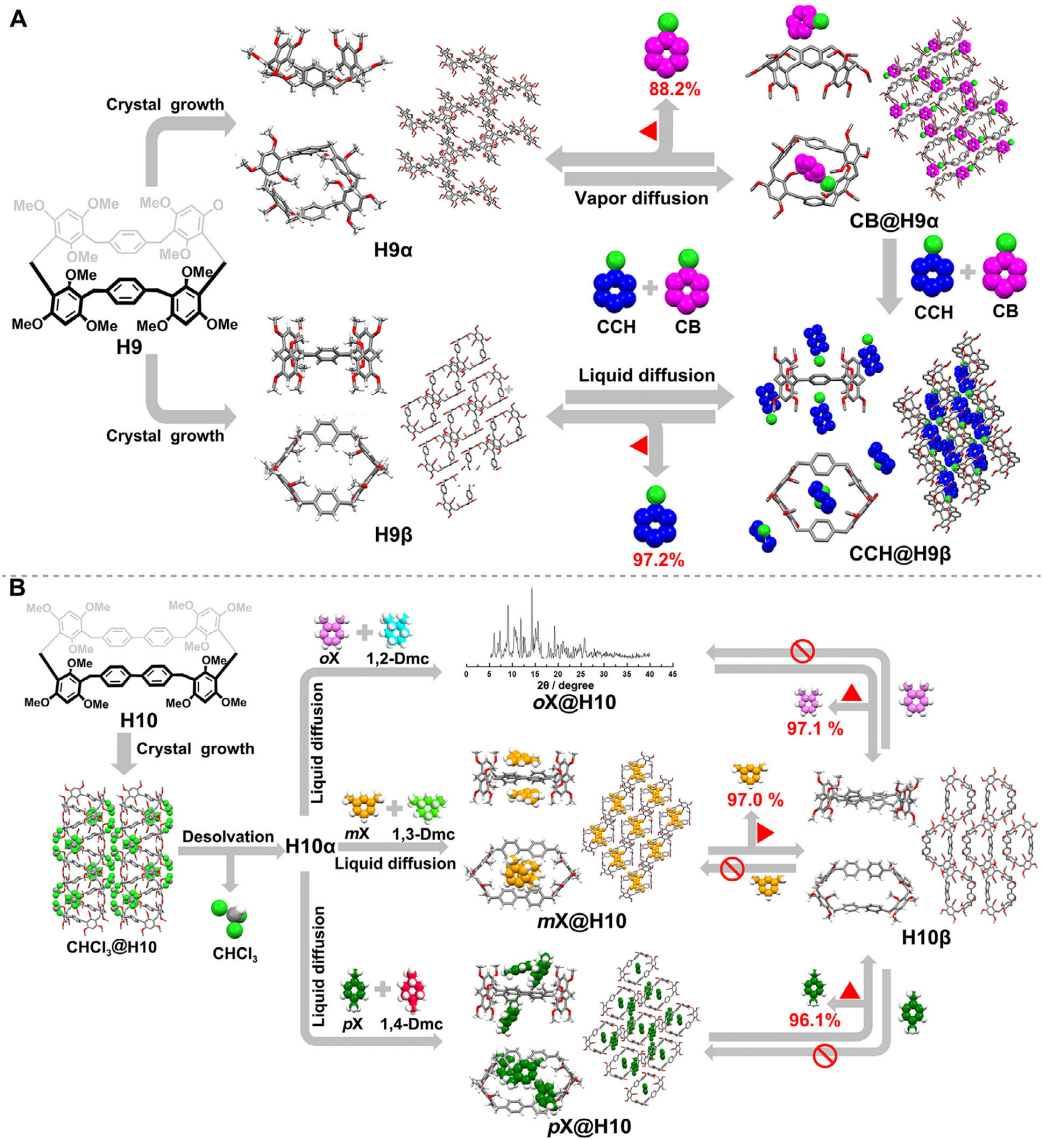
(A) Schematic illustration of the selective separation of **CB** and **CCH** using two types of **H9**‐based NACs, that is **H9α** and **H9β**. Reproduced with permission [57]. Copyright 2019, American Chemical Society. (B) Schematic representation of the separation of dimethylbenzene from its corresponding hydrogenation product using **H10α**. Reproduced with permission [61]. Copyright 2020, John Wiley and Sons.

Inspired by the synthetic methodologies of leaning pillararenes, Wu and Yang introduced a novel macrocyclic arene named geminiarene (**H9**) in 2019 [57]. **H9** integrates the monomeric linkage patterns of both pillararenes and calixarenes, featuring a hybrid *ortho*–*meta* connection mode. In the solid state, **H9** retains the structural characteristics of both pillararenes and calixarenes, with each conformation exhibiting distinct binding affinities and selectivities toward identical guests. Moreover, different guests can precisely induce and stabilize different molecular conformations of **H9**. Taking advantage of this dual conformational feature, they developed two types of NACs based on **H9** (**H9α** and **H9β**) (Figure 7A). **H9α**, crystallized in toluene, effectively separates chlorobenzene (**CB**) from a 1:1 (v:v) CB/chlorocyclohexane (CCH) mixture via solid–vapor phase adsorption with over 88% purity. This process involves a phase transition from **H9α** to a **CB**‐loaded form (**CB@H9**). Conversely, **H9β**, crystallized in dichloromethane, selectively separates CCH from the same mixture through solid–liquid phase adsorption with over 98% purity, transitioning to a **CCH**‐loaded form (**CCH@H9**). Notably, **H9α** exhibits a successive crystalline phase transformation from **H9α** to **CB@H9**, followed by **CB@H9** to **CCH@H9** upon exposure to the **CB**/**CCH** mixture, showcasing dual adsorption selectivity toward **CB** and **CCH**. This study marks the first instance of macrocycle‐based NACs demonstrating transformable/dual selectivity for molecular separation.

Based on the aforementioned work, in 2020, they successfully designed and synthesized an extended version of geminiarene, namely elongated‐geminiarene (**H10**), by replacing the benzene ring units in **H9** with biphenyl [61]. They subsequently demonstrated that NACs of **H10** (**H10α**) were effective for separating industrially significant mixtures of aromatic and cyclic aliphatic compounds, showing a preference for dimethylbenzene over its hydrogenation products (Figure 7B). Specifically, **H10α** selectively adsorbed ***o*X**, ***m*X**, and ***p*X** from mixtures (v:v = 1:1) of ***o*X**/1,2‐dimethylcyclohexane, ***m*X**/1,3‐dimethylcyclohexane, and ***p*X**/1,4‐dimethylcyclohexane with purities of 97.1%, 97.0%, and 96.1%, respectively. This led to structural transformations of **H10α** into xylene‐loaded forms, namely ***o*X@H10**, ***m*X@H10**, and ***p*X@H10**. In contrast to NACs based on other macrocycles, the removal of xylene from the loaded crystals transformed them into a guest‐free, thermodynamically favored new phase (**H10β**). However, the capability for adsorption and separation was completely lost, potentially due to its limited structural adaptability in the solid state.

## 8 Bowtiearene and Rhombicarene‐Based NACs

**FIGURE 8:**
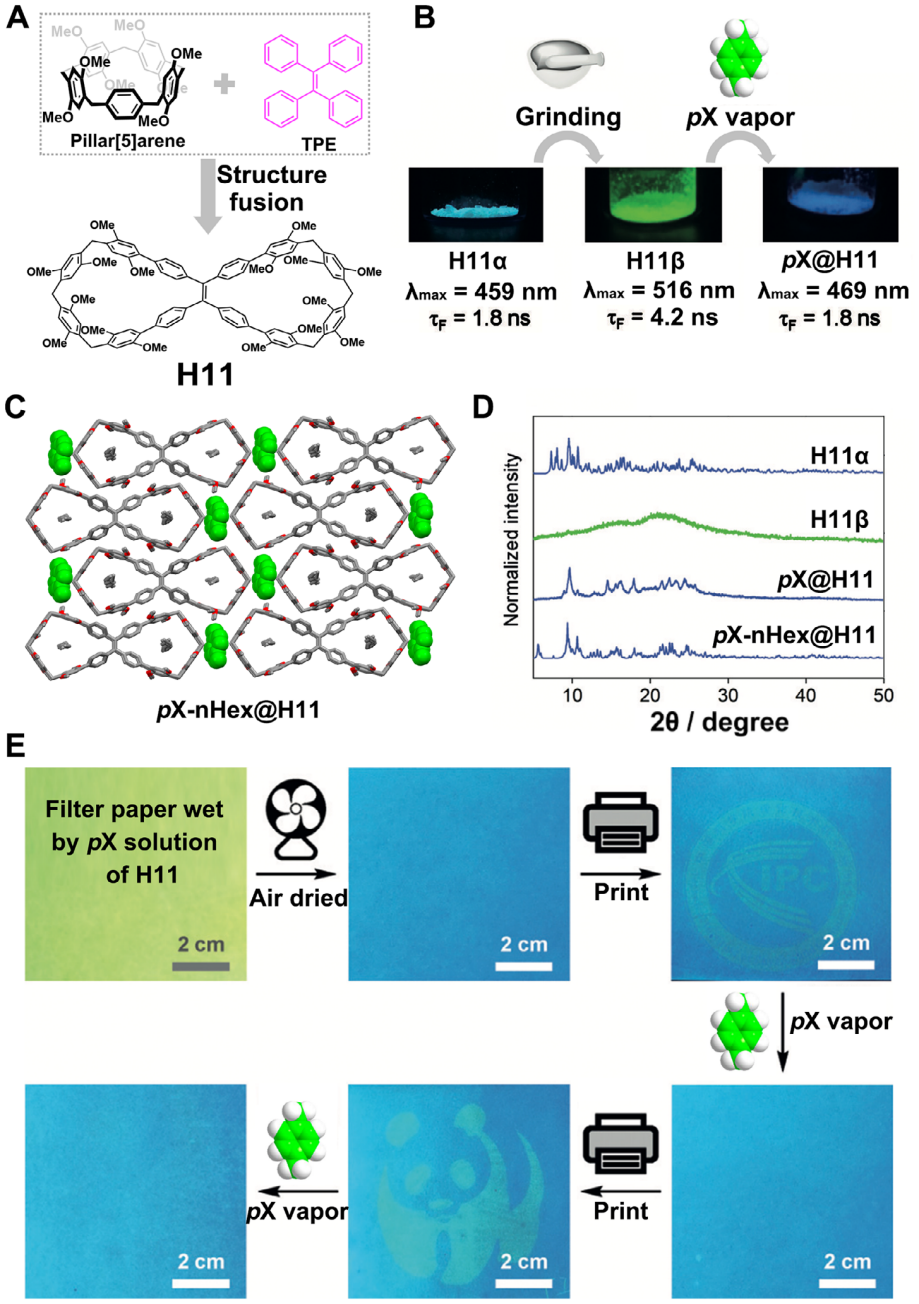
(A) The design and structural feature of **H11**. (B) Fluorochromic behavior of **H11** in response to mechanical force and ***p*X** vapor. (C) Single crystal structure of ***p*X‐nHex@H11** with ***p*X** and **nHex** showing in space‐filling and capped stick modes, respectively. (D) PXRD patterns of **H11α**, **H11β**, ***p*X@H11** and simulated pattern from ***p*X‐nHex@H11**. (E) Recyclable stimuli‐responsive fluorochromism of a piece of filter paper loaded with **H11** under 365 nm UV light. Reproduced with permission [68]. Copyright 2020, John Wiley and Sons.

Bowtiearene (**H11**) is a novel fluorescent macrocyclic host derived from pillar[n]arenes, introduced by Cong and coworkers in 2020 [68]. It features a distinctive bowtie‐shaped molecular structure composed of two pillar[5]arene backbones connected by a tetraphenylethylene unit (Figure 8A). Leveraging its inherent fluorescent substructure and dual binding cavities, NACs of **H11** (**H11α**) exhibited fluorochromism in response to mechanical force and vapor exposure. Specifically, **H11α** showed a noticeable red shift in emission from 459 to 516 nm and an increased emission lifetime from 1.8 to 4.2 ns upon grinding into powder (**H11β**), which reverted back to 469 nm with a lifetime of 1.8 ns after exposure to ***p*X** vapor (Figure 8B). Single‐crystal structure analysis and PXRD synergistically confirmed that these stimuli‐responsive shifts resulted from the destruction and reconstruction of molecular arrangements in **H11** crystals, transitioning from crystalline **H11α** to amorphous **H11β**, and then to a crystalline ***p*X**‐containing form (***p*X@H11**) (Figure 8C,D). To verify the reversibility of the stimuli‐responsive fluorochromism, filter paper loaded with ***p*X@H11** was prepared by soaking it in a dilute ***p*X** solution of **H11**, followed by air drying. Notably, delicate figures can be drawn on the paper using a dot matrix printing process and visualized under 365 nm UV light, which can be erased by fuming with ***p*X** vapor (Figure 8E). Moreover, this “printing and erasing” process can be repeated many times without loss of performance, indicating excellent reversibility and stability.

**FIGURE 9:**
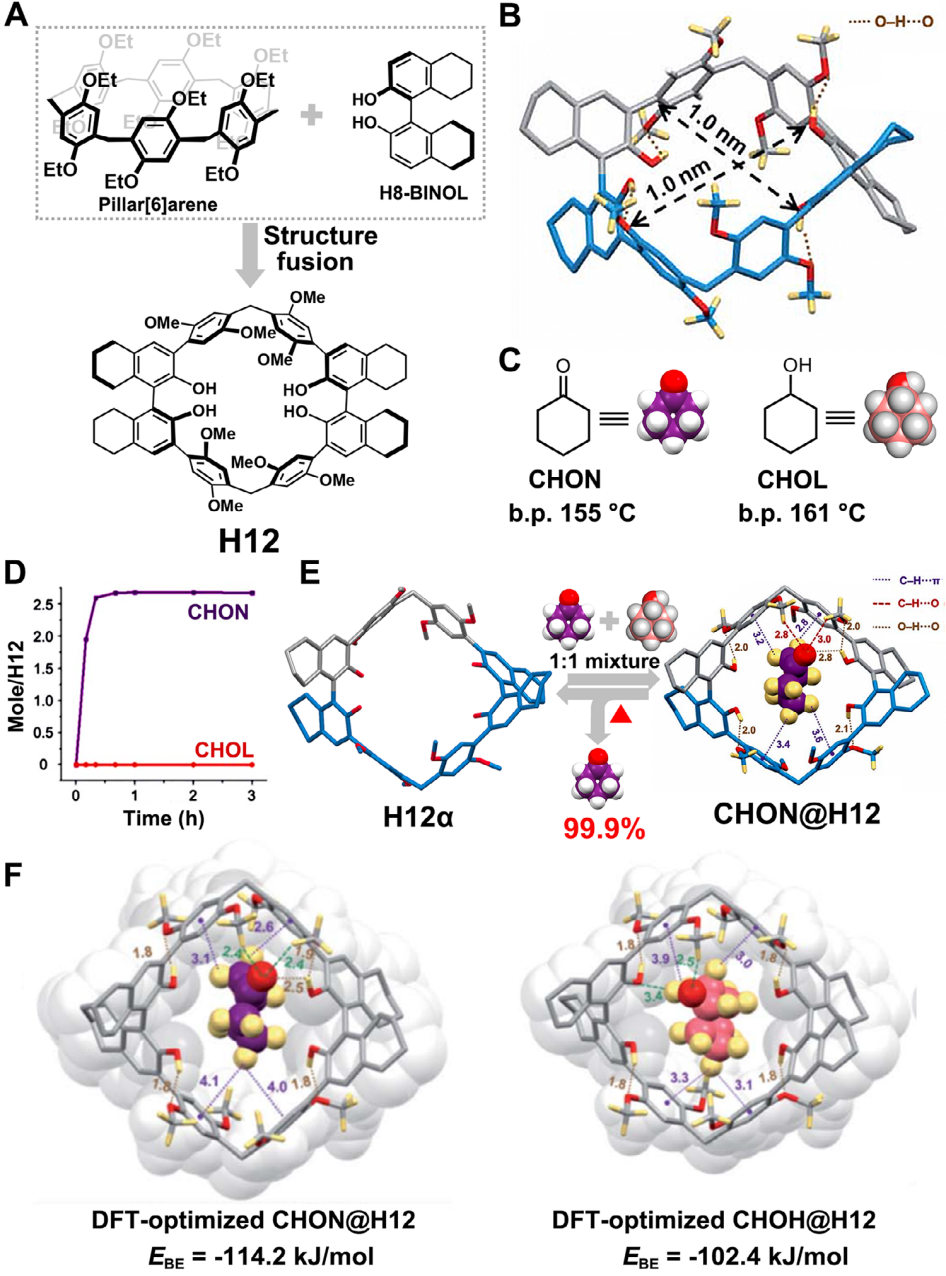
(A) The design and structural feature of **H12**. (B) Single crystal structure of **H12**. (C) Chemical structures and cartoon representations of **CHON** and **CHOL**. (D) Time‐dependent **H12α** solid‐vapor adsorption plots for single‐component **CHON** and **CHOL** vapors. (E) Schematic illustration of the separation of **CHON** and **CHOL** using **H12α**. (F) DFT‐optimized structures for **CHON@12** and **CHOLN@12**. Reproduced with permission [62]. Copyright 2021, Royal Society of Chemistry.

In 2021, the same group introduced another novel macrocyclic arene, rhombicarene (**H12**), which combines features of pillararene and octahydrobinaphthol (H8‐BINOL) (Figure 9A) [62]. **H12** was synthesized from H8‐BINOL through a three‐step synthetic method with 1,4‐cyclohexanedione serving as the template reagent during macrocyclization. Single crystal structure analysis revealed its rhombic‐shaped and nanometer‐sized cavity structure, where the OH groups on the H8‐BINOL subunits point inside the cavity and interact with adjacent *para*‐dimethoxy subunits via intramolecular hydrogen bonds (Figure 9B). Subsequently, they demonstrated that NACs of **H12** (**H12α**) could effectively function as adsorbents for the selective adsorption separation of cyclohexanol (**CHOL**) and cyclohexanone (**CHON**) (Figure 9C). Single‐component solid–vapor adsorption experiments indicated that **H12α** could adsorb **CHON** over time, while the uptake of **CHOL** was negligible (Figure 9D). Upon exposure to an equal volume mixture of **CHON** and **CHOL**, **H12α** exhibited exceptional selectivity by adsorbing **CHON** with near‐perfect purity exceeding 99.9%, resulting in a structural transformation into a **CHON**‐loaded form (**CHON@H12**) (Figure 9E). The crystal structure of **CHON@H12** revealed that the adsorbed **CHON** molecules were stably encapsulated within the cavities of **H12**, forming 1:1 host–guest complexes stabilized by multiple O─H··O, C─H··O, and C─H··π interactions. Moreover, DFT calculations demonstrated that the binding energy between **CHOL** and **H12** was lower than that between **CHON** and **H12** (Figure 9F), further confirming the adsorptive selectivity toward **CHON** from a theoretical perspective. Importantly, **H12α** can also efficiently remove trace amounts of **CHON** from **CHOL** to produce high‐purity **CHOL**, highlighting its practical utility for CHOL purification.

## 9 Phenanthrene[2]Arene‐Based NACs

**FIGURE 10:**
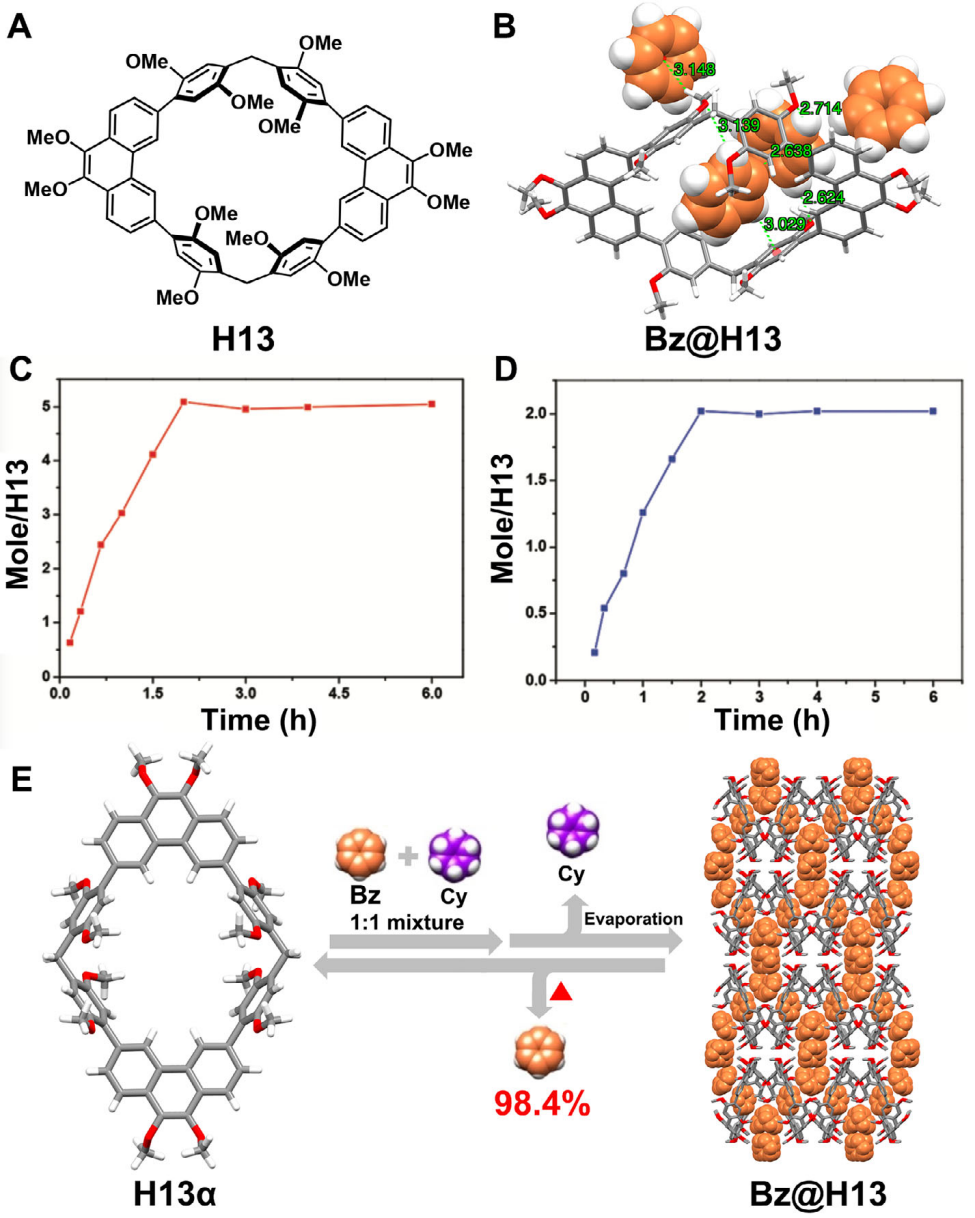
(A) Chemical structure of **H13**. (B) Single crystal structure of **Bz@H13**. Time‐dependent solid–vapor adsorption plots of **H13α** for single‐component (C) **Bz** and (D) **Cy** vapors. (E) Schematic representation of a proposed approach to the separation of **Bz** over **Cy** using **H13α**. Reproduced with permission [66]. Copyright 2022, Royal Society of Chemistry.

Phenanthrene[2]arene (**H13**), a novel macrocyclic arene combining the structural features of pillararene and phenanthrene, was first introduced by Zeng and coworkers in 2022 (Figure 10A) [66]. Single crystals of **H13** suitable for X‐ray diffraction were obtained by slow evaporation of its solution in **Bz**. Crystal structure analysis revealed that one **H13** molecule can accommodate four **Bz** molecules to form a 1:4 host–guest assembly (**Bz@H13**), stabilized by multiple C─H··O and C─H··π interactions (Figure 10B). Due to its high binding capacity towards **Bz**, they subsequently investigated the separation of **Bz** and **Cy** using NACs based on **H13** (**H13α**). Single‐component solid‐vapor sorption experiments confirmed that **H13α** could adsorb both **Bz** and **Cy** over time, with saturation adsorption capacities determined to be five **Bz** and two **Cy** molecules per **H13** molecule, respectively (Figures 10C,D). However, two‐component competitive sorption experiments using an equimolar mixture of **Bz** and **Cy** demonstrated only moderate adsorption selectivity for **Bz**, suggesting limitations in directly separating **Bz**/**Cy** mixtures using **H13α**. Interestingly, the purity of adsorbed Bz molecules could be significantly improved to 98.4% by allowing the adsorbed material to stand at room temperature for 4 h, facilitating the evaporation of loosely bound Cy molecules (Figure 10E). This approach represents a novel strategy for obtaining high‐purity chemicals using NACs, even without exceptional adsorptive selectivity. Shortly after, they also demonstrated the adsorption and separation of xylene isomers using **H13α**, which exhibited a preference for ***p*X** over ***m*X** [63].

## 10 Prism[5]Arene and Pagoda[5]Arene‐Based NACs

**FIGURE 11:**
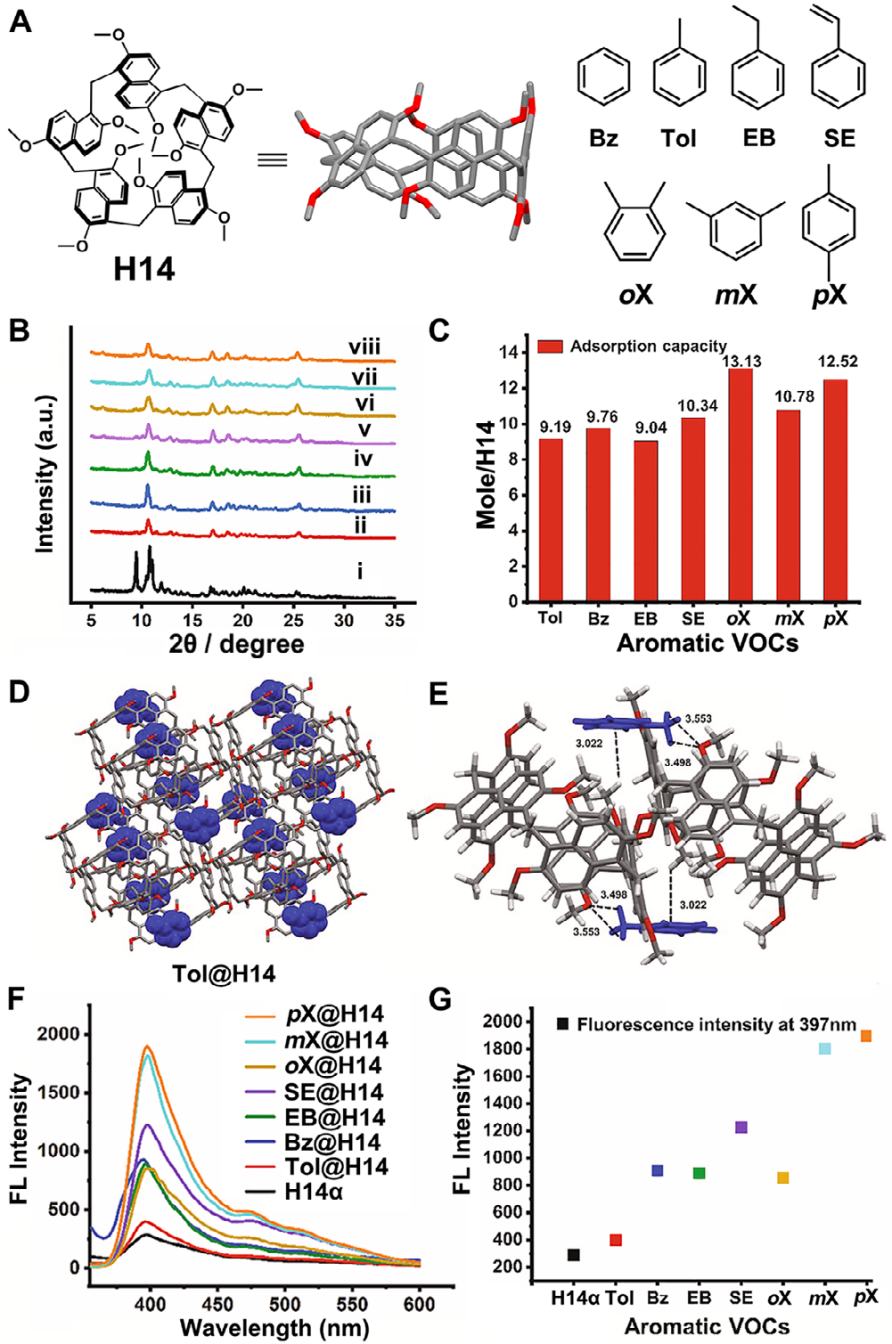
(A) Chemical structures of the macrocyclic host **H14** and the selected aromatic VOCs. (B) PXRD patterns of **H14**: (i) **H14α**; after adsorption of (ii) **Tol**, (iii) **Bz**, (iv) **EB**, (v) **SE**, (vi) ***o*X**, (vii) ***m*X**, and (viii) ***p*X**. (C) The adsorption capacity of **H14α** for different aromatic VOCs after 24 h. (D) Single crystal structure of **Tol@H14**. (E) The interactions between **Tol** and **H14** in **Tol@H14**. (F) The solid‐state fluorescence of **H14α** before and after adsorption of different VOCs. (G) The relative fluorescence intensity of **H14α** at 397 nm before and after adsorption of different VOCs. Reproduced with permission [64]. Copyright 2022, Elsevier.

Prism[n]arenes, a novel class of macrocyclic arenes composed of five or six 1,5‐naphthalene subunits bridged by methylenes, were first introduced by Gaeta and coworkers in 2020 [119, 120]. They can be synthesized directly via a one‐pot phenol–formaldehyde condensation using structurally complementary ammonium‐templating agents. In 2021, prism[5]arene (**H14**), characterized by a preorganized π‐electron‐rich cavity and a well‐defined prism‐like structure, was developed by Chang and coworkers as NACs for the capture and detection of volatile organic compounds (VOCs) (Figure 11A) [64]. Specifically, NACs of **H14** (**H14α**) effectively adsorb multiple aromatic VOCs, including **Bz**, toluene (**Tol**), ethylbenzene (**EB**), styrene (**SE**), and xylene isomers, accompanied by guest‐induced crystalline phase transformations from **H14α** to various VOC‐loaded forms (Figure 11B). The adsorption capacities for these VOCs were determined to be approximately 10 equivalents of the molar amount of **H14α**, surpassing those of commonly used activated carbon and molecular sieves under comparable conditions (Figure 11C). Single‐crystal structure analysis revealed that the adsorbed VOC molecules were not located within the cavities of **H14**, but instead occupied self‐assembled pores formed by adjacent **H14** molecules (Figure 11D,E). Interestingly, this binding mode inhibits molecular aggregation of **H14** in the solid state, thereby enhancing the solid‐state fluorescence of **H14α** to varying degrees after absorption of different VOC species (Figure 11F,G). Thus, the intrinsic fluorescence properties combined with the “outside” binding mode enable **H14α** to exhibit capabilities in the simultaneous adsorption and detection of multiple types of aromatic VOCs, highlighting a novel approach in constructing multifunctional NACs based on unfunctionalized/pristine macrocycles.

**FIGURE 12:**
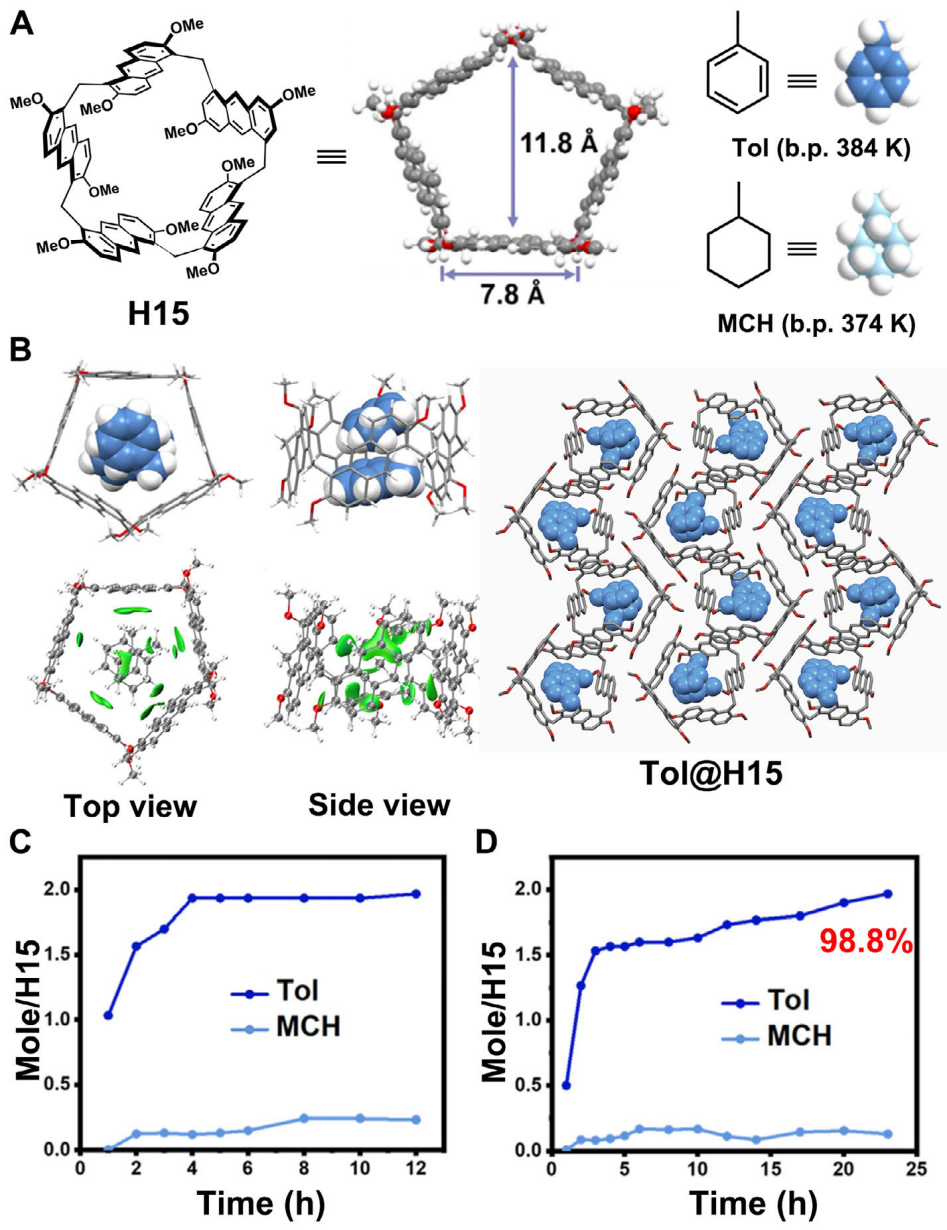
(A) Chemical structures and cartoon representations of **H15**, **Tol**, and **MCH**. (B) Single crystal structure and intermolecular binding isosurface of **Tol@H15**. (C) Time‐dependent solid–vapor adsorption plots of **H15α** for single‐component **Tol** and **MCH** vapors. (D) Time‐dependent solid–vapor adsorption plots of **H15α** for a 50:50 v/v **Tol**/**MCH** mixture vapor. Reproduced with permission [69]. Copyright 2024, Royal Society of Chemistry.

Pagoda[n]arenes, a novel class of anthracene‐based chiral macrocyclic arenes characterized by deep π‐electron‐rich cavities and pagoda‐like structures, were first introduced by Chen and coworkers in 2020 [121]. They can be conveniently obtained through a one‐pot TFA‐catalyzed condensation using 2,6‐dimethoxylanthracene as the building block. In 2024, pagoda[5]arene (**H15**), composed of five identical anthracene subunits bridged by methylenes, was developed as NACs by Huang et al. and successfully applied in the selective separation of **Tol** and methylcyclohexane (**MCH**) (Figure 12A) [69]. In their study, it was demonstrated that **H15** exhibits excellent binding affinity towards **Tol** in chloroform solution, where the host–guest binding stoichiometry was determined to be 1:2, with association constants estimated at (2.03 ± 0.13) × 10^3^ M^−1^ and (1.79 ± 0.02) × 10^2^ M^−1^ for *K*_a1_ and *K*_a2_, respectively. In contrast, no noticeable host–guest interactions were observed between **H15** and **MCH** in chloroform. Consistent with the solution‐phase molecular recognition study, single crystals of **Tol**‐loaded **H15** (**Tol@H15**) also revealed a 1:2 host–guest assembly, where two **Tol** molecules were simultaneously encapsulated within one **H15** host, stabilized by multiple C─H⋯π interactions (Figure 12B). Subsequent single‐component solid‐vapor sorption experiments confirmed that **H15α** could adsorb **Tol** vapor over time with a saturation adsorption capacity of two **Tol** molecules per **H15** host (Figure 12C). In comparison, only a small amount of **MCH** (0.24 mol mol^−1^ **H15** molecules) was adsorbed at the saturation point. Upon exposure to a 1:1 (v:v) mixture of **Tol** and **MCH**, **H15α** exhibited excellent selectivity by adsorbing **Tol** with a purity of 98.8%, accompanied by a phase transition to **Tol@H15** (Figure 12D). Furthermore, solid–vapor sorption experiments involving a series of **Tol**/**MCH** mixtures with different volumetric ratios confirmed that **H15α** maintained a relatively high adsorptive selectivity towards **Tol** even when exposed to a 1:10 (v:v) **Tol**/**MCH** mixture. These findings underscore the outstanding molecular discrimination ability and potential practical utility of **H15α** for the purification of **Tol** or **MCH**.

## 11 Conclusion and Perspective

In summary, this review summarizes the development of NACs based on novel macrocyclic arenes inspired by pillararene and calixarene structures, encompassing biphen[3]arene, tiara[5]arene, hybrid[3]arene, leaning tower[6]arene, geminiarene, elongated‐geminiarene, bowtiearene, rhombicarene, leggero pillar[5]arene, phenanthrene[2]arene, prism[5]arene, and pagoda[5]arene. These macrocyclic arenes, known for their custom‐designed structures, not only expand the toolkit of macrocycle‐based NACs but also address specific scientific and application challenges that traditional pillararene‐based NACs struggle with. For instance, NACs based on tiara[5]arene and hybrid[3]arene address the application limitations of traditional pillararene‐based NACs in selectively adsorbing and separating mixed systems of benzene and cyclohexane. Additionally, bowtiearene and prism[5]arene demonstrate intrinsic fluorescence properties, enabling the construction of versatile NACs with stimuli‐responsive fluorochromism and selective adsorption functionalities using unfunctionalized macrocycles.

However, compared to the well‐established pillararene‐based NACs, research on non‐pillararene NACs remains in its early stages, with several challenges that need to be addressed:

- In terms of application universality, these NACs based on novel macrocyclic arenes selectively address the separation of specific hydrocarbons or classes thereof. However, their applicability is less universal compared to traditional pillararene‐based NACs. Therefore, instead of solely expanding the library of macrocyclic entities to construct diverse NACs, the strategic design or discovery of supramolecular hosts that exhibit broad applicability across various adsorption and separation scenarios could hold greater significance and value for the future development of NACs.
- In terms of preparation, many novel NACs involve complex multi‐step syntheses, whereas pillararenes benefit from simpler, one‐step methods. Optimizing synthetic routes to improve efficiency, scalability, and yield is critical for advancing these materials.
- In terms of functionality, while these new NACs may offer novel features like stimuli‐responsiveness and dual adsorption selectivity that are not directly achievable with unfunctionalized pillararenes, key challenges like substrate recovery and resistance to vapor mass transfer remain unresolved. Addressing these issues is necessary for improving the practical performance of NACs.

Looking forward, future research should focus on several key areas. Understanding the influence of functional groups on crystallinity and adaptability will be essential for optimizing NAC performance. Additionally, designing macrocycles that balance structural flexibility with thermal stability will improve their recyclability. Modulating intermolecular weak interactions to enhance selectivity and developing scalable, cost‐effective synthesis methods are also critical for advancing these materials.

In conclusion, while NACs based on novel macrocyclic arenes represent a significant advancement in supramolecular chemistry, there are still several challenges to address. By overcoming issues related to synthesis, functionality, and universality, the full potential of these materials can be realized. With continued research, these NACs could lead to significant breakthroughs in various applications, such as selective adsorption, gas separation, and sensing, offering a complementary alternative to traditional crystalline materials in both industrial and environmental contexts.

## Conflicts of Interest

The authors declare no conflicts of interest.
